# Enhanced Recovery After Surgery: Exploring the Advances and Strategies

**DOI:** 10.7759/cureus.47237

**Published:** 2023-10-17

**Authors:** Shubhi N Jain, Yashwant Lamture, Malay Krishna

**Affiliations:** 1 Medicine, Jawaharlal Nehru Medical College, Datta Meghe Institute of Higher Education and Research, Wardha, IND; 2 Surgery, Jawaharlal Nehru Medical College, Datta Meghe Institute of Higher Education and Research, Wardha, IND

**Keywords:** intraoperative care, eras protocols, enhanced recover after surgery, preoperative assessment and risk management, postoperative nutrition care, surgical outcome

## Abstract

Enhanced recovery after surgery (ERAS) has emerged as a paradigm-shifting approach in perioperative care, aimed at optimizing patient outcomes, accelerating recovery, and minimizing hospital stays. This review delves into the latest advances and strategies within the field of ERAS, encompassing a comprehensive examination of preoperative, intraoperative, and postoperative interventions. By analyzing an array of clinical studies, meta-analyses, and implementation experiences, this review highlights the multifaceted elements contributing to the success of ERAS programs. Key components such as preoperative patient education, minimally invasive surgical techniques, tailored anesthesia protocols, judicious fluid management, optimized pain control, early ambulation, and structured nutritional support are thoroughly explored. Furthermore, the review delves into the intricacies of ERAS implementation across diverse surgical specialties, emphasizing the significance of multidisciplinary collaboration, protocol customization, and sustained quality improvement initiatives. The analysis not only showcases the tangible benefits of ERAS, including reduced complication rates, shortened hospital stays, and enhanced patient satisfaction, but also underscores the challenges and barriers that medical professionals encounter during program adoption. By synthesizing the current state of ERAS research and practice, this review provides clinicians, administrators, and researchers with valuable insights into the evolving landscape of perioperative care, fostering a deeper understanding of ERAS as a holistic approach that transcends traditional surgical pathways.

## Introduction and background

Enhanced recovery after surgery (ERAS) is a worldwide drive in the field of surgery focusing on improvement in quality of care, before, during, and after surgery. While ERAS has been linked with notable enhancements in clinical outcomes and cost reductions across various surgical fields, there are several areas of potential exploration and difficulties that warrant additional discussion [1-3]. The concept of fast-track surgery or ERAS was formulated by Professor Henrik Kehlet during the mid-1990s for individuals undergoing colorectal surgery. This approach led to a notably reduced duration of postoperative hospitalization [4]. Following this, the ERAS Society was founded in 2010, and since then, guidelines have been released for various surgical procedures such as colorectal surgery, bariatric surgery, gastrectomy, liver surgery, and gynecologic oncology [4,5]. The implementation of the ERAS process requires a collaborative group comprising surgeons, an ERAS coordinator (frequently a physician assistant or nurse), anesthetists, and personnel from the departments responsible for the care of surgical patients [6]. Numerous components of ERAS care, including the adoption of minimally invasive surgical methods and the avoidance of fasting, work to reduce the body's stress response to surgery and support the preservation of homeostasis. This approach aids in preventing significant catabolism and the resulting depletion of body protein, strength, and overall function in the patient [6-8].

The fundamental principles of ERAS encompass comprehensive preoperative counseling, elimination of bowel preparation, avoidance of sedative premedication, absence of preoperative fasting, intake of preoperative carbohydrates, personalized anesthesiology, controlled perioperative intravenous fluid administration, non-opioid pain control, selective usage of drains and nasogastric tubes, early initiation of both postoperative nourishment and prompt removal of the urinary catheter, and mobilization [9-11].

Among the seven ERAS protocols examined, same-day discharge was facilitated by five. Additionally, three of these protocols incorporated minimally invasive techniques for spine surgery, which prioritized reduced blood loss and muscle trauma. Furthermore, one ERAS protocol introduced the concept of awake surgery. The study underscored the importance of avoiding extended preoperative fasting, as this has been linked to detrimental impacts on muscle catabolism. The researchers emphasized that up to six hours before surgery, patients are encouraged to consume a light meal and up to two hours before the procedure, clear liquids can be consumed. The study also advocated for carbohydrate supplementation. During the intraoperative phase, the recommendation is to proactively progress the patient's diet, enabling them to eat and drink within hours following the surgery [12-14].

## Review

Methodology

A systematic search was undertaken through PubMed and Scopus in May 2023 using keywords such as "enhanced recovery after surgery" and "intraoperative complications" (((enhanced recovery after surgery [Title/Abstract]) OR (ERAS [Title/Abstract])) OR ("enhanced recovery after surgery " [MeSH Terms]) AND (("intraoperative complications " [Title/Abstract]) OR ("intraoperative complications" [MeSH Terms]). We additionally searched for key references from bibliographies of the relevant studies. The search was updated in July 2023.

**Figure 1:**
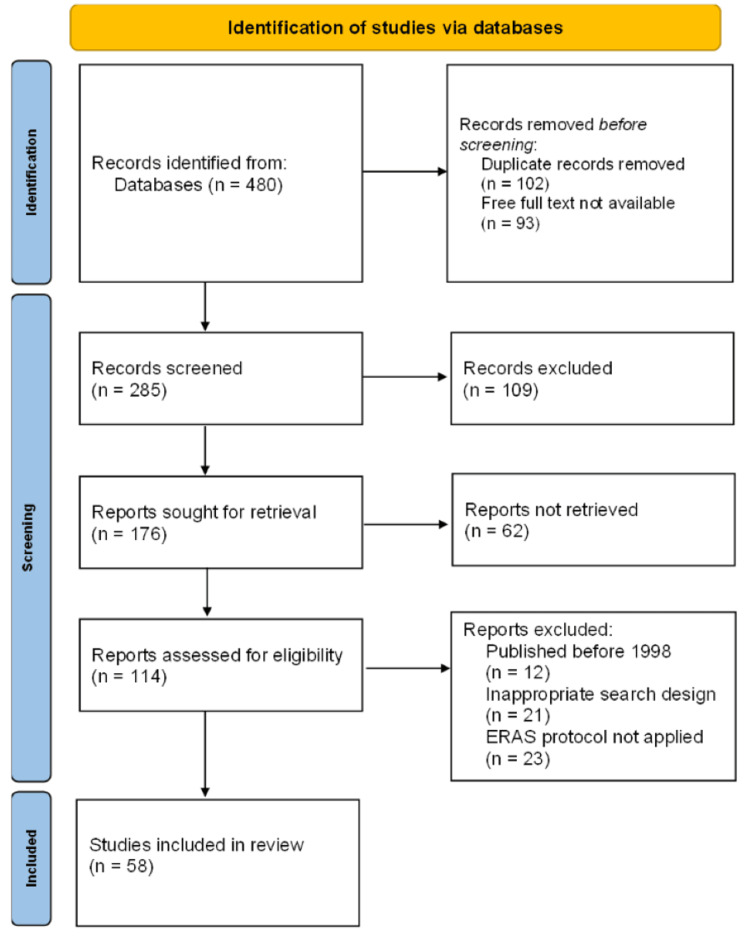
PRISMA flow diagram of search strategy PRISMA, Preferred Reporting Items for Systematic Reviews and Meta-Analyses; ERAS, enhanced recovery after surgery

One reviewer independently monitored the retrieved studies against the inclusion criteria, in the beginning, based on the title and abstract and then on full texts. Another reviewer also reviewed approximately 20% of these studies to validate the inclusion of studies. A Preferred Reporting Items for Systematic Reviews and Meta-Analyses (PRISMA) flow diagram is used to demonstrate the number of studies included (Figure 1).

Enhanced recovery after surgery

**Figure 2:**
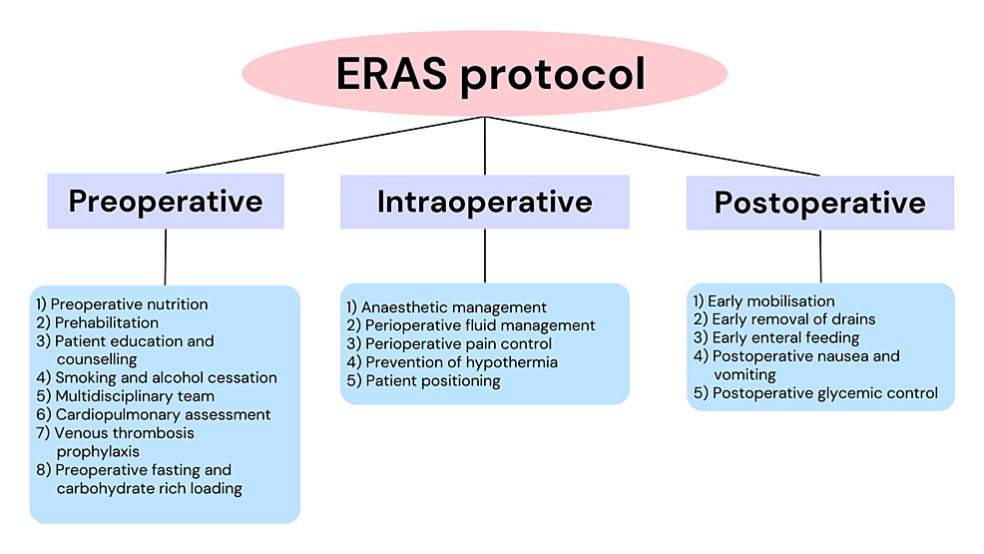
Components of the ERAS protocol ERAS, enhanced recovery after surgery Image credit: Shubhi Jain [17]

ERAS represents a comprehensive, collaborative strategy aimed at enhancing the care of patients through the integration of techniques that are evidence based. The interventions within ERAS protocols are founded on the premise that by managing pain, optimizing fluid balance, promoting early mobility, and providing proper nutrition, patient outcomes can be enhanced. The underlying concept is to prevent catabolism and immune dysfunction. Distinct ERAS protocols exist for various surgical domains, all united by the common objective of reducing physiological stress experienced by patients [15,16]. The components of ERAS include preoperative, intraoperative, and postoperative components (Figure 2).

Preoperative components

Preoperative Nutrition

Differing from preoperative weight loss programs that are dictated by insurance, a typical approach before bariatric surgery involves a two- to four-week phase of adhering to a low-calorie diet or a very low calorie diet (VLCD). This protocol has demonstrated the ability to decrease the volume of the liver and the recognized surgical complexity from a surgeon's perspective [18,19]. Moreover, a randomized controlled trial (RCT) revealed an enhancement in sensitivity for whole-body insulin when VLCD was taken for a couple of weeks [20].

Prehabilitation

Several RCTs, which are small-scale, have investigated the impact of prehabilitation in individuals slated for bariatric surgery. An exercise regimen of 12 weeks encompassing endurance training, demonstrated a correlation with a decrease in weight, enhanced overall physical fitness, and showed improvements in cardiometabolic risk factors [21].

A training regimen of six weeks preoperatively also displayed a connection to sustained enhancements in physical activity six months following the surgery. Moreover, targeted inspiratory muscle training before surgery led to immediate (within 12 hours) postoperative improvements in oxygenation and an increase in the strength of the muscles of inspiration [22].

This review study focused on prehabilitation conducted several months prior to surgery. The prehabilitation approach encompassed a rigorous exercise routine initiated two months before the surgical procedure, the use of pain-controlling medications, and the consumption of protein drinks on the day preceding the operation. Implementing early mobilization protocols led to a decrease in the incidence of complications and morbidity, such as issues like respiratory problems, pneumonia, deep venous thrombosis, pulmonary embolism, urinary tract infections, sepsis, and infections. Additionally, it contributed to a reduction in the average length of stay. The review's conclusion highlighted that patients who underwent prehabilitation and early ambulation experienced improved outcomes across various postoperative measures. This included a shorter average hospital stay (reduced from seven days to five days) and greater postoperative patient satisfaction [23].

Patient Education and Counseling

Patients who are scheduled for bariatric surgery should be thoroughly educated about the significant lifestyle changes that come with the procedure. A preoperative educational program is frequently advised to establish accurate expectations, minimize anxiety, lower the risk of wound complications, alleviate postoperative pain, and reduce the length of hospital stay [24]. In a meta-analysis that encompassed patients undergoing cancer surgery, it was observed that education not only decreased anxiety and healthcare expenses but also enhanced patients' knowledge and satisfaction [25].

Smoking and Alcohol Cessation

Discontinuing smoking for a minimum of four to eight weeks before surgery has been proven to lower postoperative complications following non-bariatric surgeries, particularly in terms of wound and cardiovascular issues. In the context of bariatric surgery, smoking is linked to an elevated risk of marginal ulcers, infectious complications, and respiratory problems [26,27].

Excessive alcohol consumption is known to heighten the likelihood of postoperative complications, primarily those tied to infections and wound healing. The intricate nature of necessary behavioral changes, coupled with an increased vulnerability to postoperative alcohol overindulgence and dependency, particularly in patients with previous substance abuse histories, forms the foundation for current recommendations. These recommendations advocate for a documented period of alcohol abstinence lasting one to two years for patients who have previously struggled with excessive consumption [28,29].

Multidisciplinary Team

**Figure 3:**
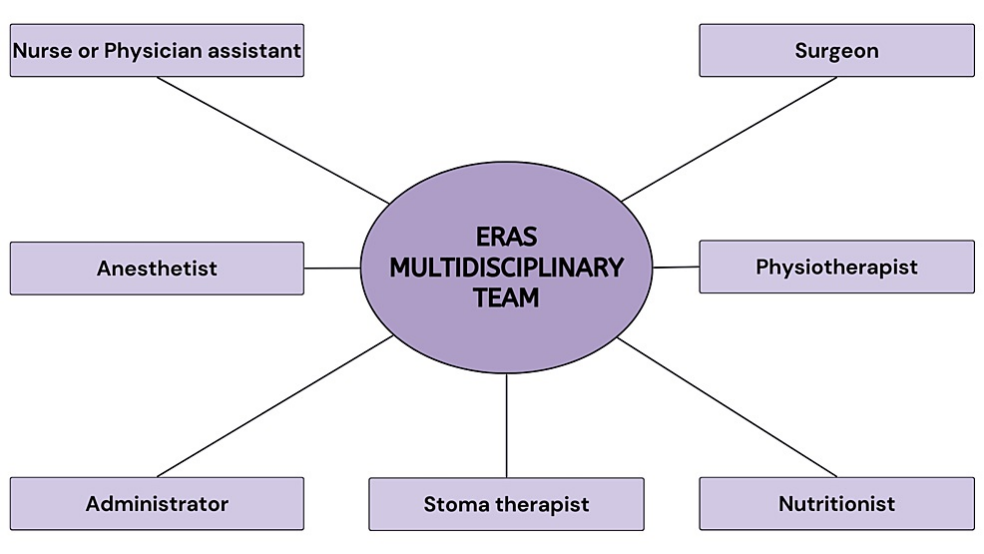
Members forming an ERAS multidisciplinary team ERAS, enhanced recovery after surgery Image credit: Shubhi Jain [31]

The successful establishment and ongoing operation of an ERAS pathway necessitate collaborative efforts from a multidisciplinary team across the entirety of a patient's journey [30]. When undergoing formal training to implement an ERAS pathway within their hospital or unit, a multidisciplinary team is assembled. This team typically includes a dedicated nurse or physician assistant, an anesthetist, an administrator, and a surgeon. Other crucial healthcare professionals like physiotherapists, stoma therapists, or nutritionists may also be included to contribute their expertise [31]. A team leader, who could be a surgeon or an anesthetist, assumes the responsibility of setting clear objectives, fostering open communication, and supporting all team members. The overarching aim is to achieve optimal outcomes for patients [32]. In the role of a team leader, the surgeon must cultivate trust, provide motivation, and actively listen to all members of the team. This collaborative approach is pivotal in elevating the team's performance and, potentially, the patient's overall outcome [33]. A diagrammatic representation of the team members of the multidisciplinary team is shown in Figure 3.

Cardiopulmonary Assessment

In a clinical trial, out of the total observed mortality rate of 3.9%, 43% of the documented deaths were associated with insufficient cardiopulmonary function that had been detected before the surgery. Notably, no deaths were attributed to cardiopulmonary complications among patients who were deemed suitable for major abdominal surgery and standard ward care, as determined by cardiopulmonary exercise (CPX) testing [34]. This trial investigated the utility of assessing perioperative mortality and morbidity in thoracoabdominal surgeries that do not primarily involve the heart or lungs, by examining the predictive value of maximum oxygen consumption (VO_2_max) and anaerobic threshold, with both values being derived from CPX testing. A review of the existing literature encompassed nine studies that looked into either one or both of these variables across a diverse array of surgical procedures. Of the seven studies providing sufficiently detailed results on peak oxygen consumption data, six found it to be a significant predictor. Similarly, among the six studies with sufficiently detailed results on anaerobic threshold, four identified it as a significant predictor. In conclusion, peak oxygen consumption, and potentially anaerobic threshold, are credible predictors of perioperative morbidity and mortality within non-cardiopulmonary thoracoabdominal surgery. These indicators have the potential to guide the allocation of intensified care to patients at higher risk [35].

Venous Thrombosis Prophylaxis

The occurrence of venous thromboembolism following esophagectomy varies between 5% and 7%, and it is associated with a twofold increase in the risk of mortality [36]. The existing guidelines from the American College of Chest Physicians propose a dual approach to prophylaxis in high-risk patients, combining chemical measures (such as unfractionated heparin or low-molecular-weight heparin) with mechanical methods (like pneumatic compression devices or elastic stockings). Additionally, it is advised that chemoprophylaxis should commence 2-12 hours prior to the surgery and be continued for a duration of four weeks postoperatively [37].

Preoperative Fasting and Carbohydrate-Rich Loading

Programs of ERAS have brought about a shift in the guidelines regarding preoperative fasting for elective surgery. Given the absence of substantial evidence indicating added safety benefits from extended fasting periods, and considering evidence suggesting that prolonged fasting might elevate catabolic stress responses after surgery, resulting in hyperglycemia and insulin resistance, the current stance allows for the consumption of solid food up to six hours and clear liquids up to two hours before the commencement of anesthesia [38]. This change to reduced fasting periods has yielded improved patient outcomes and satisfaction due to reduced sensations of thirst and hunger, as well as a diminished risk of dehydration [39].

The protocol of ERAS suggests the inclusion of carbohydrate loading through a clear liquid intake two to three hours before surgery, provided it is suitable for the specific patient group. Carbohydrate loading has demonstrated links to enhanced insulin sensitivity and a decrease in reported hunger and thirst leading up to the surgery. Moreover, consuming carbohydrates before the surgical procedure helps counteract certain catabolic effects associated with fasting, leading to reduced infections, shorter hospital stays, and decreased overall complications [40].

Notably, a study has associated preoperative carbohydrate administration with a decrease in emergency room visits and hospital readmissions stemming from pain-related issues [41].

Intraoperative components

Anesthetic Management

There is currently no widely agreed-upon standard anesthetic protocol within the ERAS framework. A significant portion of studies, totaling 54.2%, emphasized the significance of the anesthetic protocol in their ERAS pathway. Within this subset, a substantial majority (84.6%) opted for a preferred anesthetic protocol centered around spinal techniques. Only a couple of studies indicated that a combination of general analgesia and spinal anesthesia was considered suitable [42]. Local or regional anesthesia holds a critical role in managing pain following surgery and promoting improved mobility. Specifically, local infiltration analgesia (LIA) offers advantages over nerve blocks due to its minimal impact on muscle strength and lack of blockade of motor activity. A prospective study conducted a comparison between peripheral nerve blocks, LIA, spinal anesthesia, and general anesthesia in primary total knee arthroplasty (TKA) for control of pain and early functional recovery. The findings revealed that pain relief was similar across all four groups. However, LIA demonstrated impactful benefits in terms of muscle strength and early postoperative mobility [43].

Perioperative Fluid Management

The occurrence of complications of the pulmonary system following esophagectomy is relatively frequent, and fluid overload exacerbates this further by leading to pulmonary interstitial edema. Multiple studies have demonstrated a connection between a greater cumulative fluid balance and heightened pulmonary morbidity in patients undergoing esophagectomy [44]. The "optimal" fluid management was advocated by the ERAS protocol for utilizing balanced crystalloids, with the objective of achieving weight gain of no more than 2 kg/day [45].

Perioperative Pain Control

Within ERAS protocols, the inclusion of dexmedetomidine in perioperative care has yielded notable enhancements in postoperative results. While the effectiveness of ERAS protocols has been established, there remains a major area for advancement in the realm of pain management postoperatively. Addressing this aspect would allow patients to extract maximum benefits from ERAS implementations during their postoperative phase. Elevating the management of continuing perioperative pain could result in reduced post-anesthetic recovery unit (PACU) time, earlier hospital discharge, diminished opioid usage, and an overall boost in the contentment of the patient [46].

The recommendation regarding the use of non-steroidal anti-inflammatory drugs (NSAIDs) suggests carefulness due to disquiets about potential bleeding and bone non-union, although the literature presents conflicting views on the relationship between NSAIDs and non-union as well as elevated bleeding risk. The research indicated that selective COX-2 inhibitors exhibit higher effectiveness compared to non-selective NSAIDs. Furthermore, findings demonstrated that gabapentin or pregabalin contributed to alleviating opioid-related side effects and decreased postoperative consumption of opioids [47,48].

At 4, 8, 12, and 24 hours following spine surgery, the addition of ketamine as a supplement led to decreased cumulative morphine equivalent consumption. Furthermore, at 6, 12, and 24 hours after the spine surgery, the incorporation of supplemental ketamine resulted in lower postoperative pain scores. Importantly, the use of supplemental ketamine did not increase the likelihood of patients undergoing spine surgery experiencing negative effects like cardiac events, unpleasant dreams, hallucinations, dysphoria, pruritus, postoperative nausea or vomiting, urinary retention, psychotomimetic events, sedation or respiratory depression [49].

In contrast to intravenous patient-controlled analgesia, lower postoperative pain scores on the first postoperative day (POD 1) were found to result from epidural anesthesia. On the other hand, reduced pain scores on POD 1 were observed with intrathecal morphine, but it increased the risk of respiratory depression and pruritis in patients [15,47].

Prevention of Hypothermia

Extended intra-cavity surgery proposed an elevated risk of experiencing perioperative hypothermia in individuals undergoing esophagectomy. Hypothermia, characterized by a core temperature below 36 °C, can negatively impact the metabolism of drugs, anesthesia recovery, coagulation, and the need for transfusion. It can also lead to patient discomfort and is associated with an increased risk of cardiac issues and surgical site infections. To counter this, the adoption of fluid warming and multi-modal approaches like forced-air warming is advocated by the ERAS guidelines to prevent hypothermia [50].

Patient Positioning

A notable aspect frequently addressed for spine surgeries in ERAS protocols is patient positioning. Research indicates that adjusting the patient's position to minimize abdominal pressure results in reduced blood loss. In situations where the patient's positioning is inadequate during surgery, increased abdominal pressures lead to raised pressure within the epidural venous system and the vena cava, subsequently contributing to greater bleeding [47,48].

Postoperative components

Early Mobilisation

Prompt mobilization is a pivotal aspect of ERAS protocols, counteracting the immobilization and detrimental effects of surgical stress. Early mobilization serves to mitigate the risk of postoperative complications, positively impact patient-reported outcomes, expedite the recovery of functional walking capacity, and ultimately decrease the length of hospital stays, hence reducing overall expenses of care. Challenges that can impede early mobilization include inadequate education and resource availability. Addressing these barriers through education and clinical decision-support tools can enhance compliance with ERAS mobilization recommendations and foster a culture that values perioperative physical activity. Notable advancements involve real-time feedback on mobilization levels using wearable technology and the integration of ERAS principles with exercise prehabilitation. The advantages of structured postoperative mobilization should be emphasized in future ERAS guidelines [51].

Early Removal of Drains

In the immediate postoperative phase following an esophagectomy, patients typically have several tubes and lines in place, including an arterial line, a nasojejunal or jejunostomy tube, a nasogastric tube, an intravenous catheter, a urinary catheter, a chest tube, and an epidural catheter. These tubes can complicate early mobilization efforts. If the gastric tube is not enlarged, then by POD 2 the nasogastric tube is ideally removed. Once the diuretic phase is reached or after 48 hours, the urinary catheter can be taken out, but particularly in elderly males, a 26% likelihood of re-insertion is present if the urinary catheter is removed while the epidural catheter is still in place. Chest drains can lead to limited mobility and pain. While many medical centers remove chest drains when they produce 100-150 ml of output per day, there is limited evidence to contribute to this threshold. In certain institutions, chest drains are removed when they reach 5 ml per kilogram of body weight. Usually by POD 2, in the absence of chyle leaks or air, confirmation of complete lung expansion on a chest X-ray can indicate the removal of the chest drain [52].

Early Enteral Feeds

Due to varying durations and degrees of dysphagia, patients with esophageal cancer often experience nutritional depletion, sometimes even upon diagnosis. To prevent postoperative complications arising from malnutrition and to maintain the progress made through prehabilitation, it is crucial to initiate early nutritional support after surgery. In the context of esophageal resection, enteral nutrition is preferred to parenteral nutrition. Parenteral nutrition is linked to a higher incidence of sepsis, elevated liver enzymes, and metabolic disturbances. Its usage is advisable only when enteral feeding is not practical. On POD 1, enteral nutrition should commence via a feeding jejunostomy or naso-jejunal tube. By POD 3, gradual increases in feed volumes should occur based on patients' tolerance, aiming to meet calorie requirements [45].

Postoperative Nausea and Vomiting

Prevalent and stressful complications after surgery are still postoperative nausea and vomiting (PONV). These issues can lead to escalated medical expenses, extend the duration of hospitalization, and have adverse impacts on postoperative nutritional recovery [53]. Key factors that elevate the risk of PONV comprise the use of opioids both during and after surgery, surgical procedures of longer durations, and being female [54]. In a study, the occurrence of vomiting was found to be more frequent in the group that underwent spinal anesthesia compared to the group that received general anesthesia [55]. Antiemetic medications like ondansetron when used prophylactically can mitigate or reduce the occurrence of PONV [56]. Furthermore, another medicine that is also proven to be both effective and safe for preventing PONV is dexamethasone. It has the ability to decrease the intensity and occurrence of PONV, and repeated usage of antiemetic medications can be reduced. Interestingly, within their ERAS protocols, the importance of PONV prophylaxis was underscored by barely 29.2% of the studies [57].

Postoperative Glycemic Control

Implementing an enhanced recovery protocol could potentially result in higher glucose levels preoperatively. Also, patients with diabetes mellitus type 2 who were undergoing colorectal surgery showed an increase in the rate of readmissions within 30 days. Although in diabetic patients, the actual clinical significance of temporarily elevated preoperative glucose levels remains undetermined, on the whole, the findings suggest that for diabetes mellitus patients undergoing a colorectal operation, loading carbohydrates preoperatively and adopting a protocol of enhanced recovery following surgery do not result in worse glycemic control following surgery [58].

ERAS group versus conventional group

In the ERAS group, patients were able to resume oral diet intake by the first postoperative day. This group also achieved daily mobilization exceeding six hours by postoperative days 2 to 3 and experienced bowel movements by postoperative day 2, whereas the conventional group achieved this by postoperative day 5. Notably, the ERAS group displayed comparatively lesser reductions in lung function and muscle strength. There was a decrease of two days in the median hospital stay in this group. While readmission rates increased slightly, the overall stay at the hospital remained lower in the ERAS group. Following colonic resection, the ERAS approach was associated with a reduction in postoperative complications, but no difference was observed following rectal resection. Additionally, there was a cost reduction of $5109.10 with the ERAS approach [49]. A summary of all the studies included in this review is given in Table 1.

**Table 1:** Studies included in the review ERAS, enhanced recovery after surgery; LOF, length of stay; VLCD, very low calorie diet; LCD, low-calorie diet; TLV, total liver volume; LLL, left liver lobe; VFA, visceral fat area; QoL, quality of life; IMT, inspiratory muscular training; BS, bariatric surgery; CPX, cardiopulmonary exercise; VO_2_max, maximum oxygen consumption; AT, anaerobic testing; VTE, venous thrombo-embolism; LIA, local infiltration analgesia; OT, operation theatre; PONV, postoperative nausea and vomiting; GA, general anesthesia; SA, spinal anesthesia; DM, diabetes mellitus

Authors	Year	Country of origin	Findings
Ljungqvist et al. [1]	2021	Sweden	ERAS is a worldwide drive for the betterment of the outcome of surgery.
Gillis et al. [2]	2022	Canada	Preoperative regulations can decline postoperative deaths.
Amer et al. [3]	2016	New Zealand	Preoperative loading of carbs results in a reduced stay at the hospital after surgery.
Ashok et al. [4]	2020	India	The ERAS program plays a significant role in decreasing the LOS at the hospital.
Pędziwiatr et al. [5]	2016	Poland	The advantages of minimally invasive surgery over open surgery in relation to cost savings include reduced expenses.
Ljungqvist et al. [6]	2017	Sweden	Improvements in patient prognosis have been achieved through the implementation of the ERAS protocol.
Nygren et al. [7]	2009	Sweden	Contrasting clinical results were found between the ERAS and non-ERAS groups.
Henriksen et al. [8]	2003	Denmark	A smaller decline in postoperative muscle strength was seen.
Wind et al. [9]	2006	Netherlands	Key principles of ERAS in laparoscopic surgery promote swift recovery.
Polle et al. [10]	2007	Netherlands	Fast-track modalities reduce the overall stay of patients at the hospital.
Chong et al. [11]	2019	Hongkong	ERAS protocol is reasonable and harmless for liver resection surgery.
Corniola et al. [12]	2019	Switzerland	ERAS aspects in spine surgery result in reduced cost and give a better clinical prognosis.
Dietz et al. [13]	2019	USA	Lesser complications and decreased use of opioids are seen with the ERAS protocol.
Wang and Grossman [14]	2016	USA	The minimally invasive procedure provides reduced blood loss in patients.
Naftalovich et al. [15]	2022	USA	ERAS helps minimize preoperative opioid use, contributing to improved pain management.
Elsarrag et al. [16]	2019	USA	Decreased pain after surgery and hastened gain back of function are seen following ERAS.
Melloul et al. [17]	2016	USA	Faster recovery of bowel results from oral laxatives after surgery.
Gils et al. [18]	2018	Spain	VLCD and LCD show the same effect in reducing TLV.
Bakker et al. [19]	2019	Netherlands	A decrease in TLV, LLL, and VFA is seen with LCD and omega-3 diet.
Stenberg et al. [20]	2022	Sweden	Improved whole-body insulin sensitivity is observed with a VLCD.
Marc-Hernández et al. [21]	2019	Spain	QoL is improved with preoperative physical activity.
Lloréns et al. [22]	2015	Spain	Better oxygenation in patients is seen with before-surgery IMT.
Epstein [23]	2014	USA	Early ambulation aids in decreasing the degree of complications following surgery.
Mechanick et al. [24]	2013	USA	A preoperative education program minimizes anxiety.
Waller et al. [25]	2015	Australia	Enhanced patient knowledge and satisfaction is observed with educating the patient.
Møller et al. [26]	2002	Denmark	Cessation of smoking preoperatively declines morbidity.
Haskins et al. [27]	2014	USA	Raised occurrences of shock and sepsis are seen after surgery in smokers.
Kanji et al. [28]	2019	Canada	No association of history of utilization of substance is seen with weight loss after BS.
Nath et al. [29]	2010	USA	The use of alcohol before surgery shows detrimental effects on a patient’s prognosis.
Martin et al. [30]	2018	Switzerland	ERAS protocol helps in the reduction of complications associated with surgery.
Roulin et al. [31]	2017	Switzerland	Betterment in preoperative outcome is seen with ERAS.
Onaca and Fleshman [32]	2020	USA	The surgeon's first preference is the care of the patient.
Roulin and Demartines [33]	2022	Switzerland	A multidisciplinary approach customized for patient intervention is improved upon collaboration.
Older et al. [34]	1999	Australia	Heart- and lung-related complications can be reduced with pre-surgical CPX tests.
Smith et al. [35]	2009	UK	VO_2_max and AT are important indicators for complications following surgery.
Zwischenberger et al. [36]	2016	USA	Reduction in VTE issues is observed with chemoprophylaxis.
Farge et al. [37]	2013	France	Both chemical and mechanical measures are needed to decrease VTE incidences postoperatively.
Gwynne-Jones et al. [38]	2017	New Zealand	ERAS aids in decreasing LOS and mortality.
Miller et al. [39]	2015	USA	Pre- and intraoperative fluid management is a significant ERAS protocol.
Bilku et al. [40]	2014	UK	Preoperative carb-rich fluid therapy does not show any adverse effects on the patient.
Parrish et al. [41]	2018	USA	Lesser complaints of pain were filed with the ambulatory anorectal operation.
Didden et al. [42]	2019	Netherlands	Readmission rates fall off with ERAS guidelines.
Berninger et al. [43]	2018	Germany	Better scope in the treatment plan is noted with LIA.
Kita et al. [44]	2002	Japan	The decline in the incidence of pulmonary complications is seen with adequate fluid therapy.
Low et al. [45]	2019	USA	ERAS focuses on reducing complications associated with surgery and hastening recovery after surgery.
Kaye et al. [46]	2020	USA	The use of Dexmedetomidine is beneficial in reducing pain following the OT procedure.
Alboog et al. [47]	2019	Canada	Gabapentinoids and opioids together help in reducing pain associated with surgery.
Park [48]	2000	Korea	Positioning of the patient during surgery is related to blood loss.
Pendi et al. [49]	2018	USA	Less consumption of opioids is noted.
Fu et al. [50]	2015	Japan	Fluid warming can reduce hypothermia in operative patients.
Tazreean et al. [51]	2021	Canada	Prompt mobilization is a major aspect of ERAS.
Mistry et al. [52]	2012	India	Early removal of drains is associated with early ambulation.
Mc Loughlin et al. [53]	2019	Argentina	The commonest complication of surgery is PONV.
Gan [54]	2006	USA	The use of opioids is associated with PONV.
Palanne et al. [55]	2020	Finland	In comparison to GA, SA has a lesser incidence of PONV.
Schlesinger et al. [56]	2023	Germany	Ondansetron mitigates the incidence of PONV.
Corcoran et al. [57]	2022	Australia	Dexamethasone decreases the intensity of PONV.
Cua et al. [58]	2021	USA	In DM patients, loading carbs preoperatively does not worsen the glycemic control following operation.

## Conclusions

This review highlights the remarkable strides made in the realm of enhanced recovery after surgery, shedding light on its multifaceted approaches and tangible benefits. From preoperative optimization to postoperative care, ERAS strategies have revolutionized surgical outcomes, reducing complications and hospital stays while improving patient satisfaction. By amalgamating evidence-based interventions such as prophylaxis for nausea and vomiting, optimized pain management, and early ambulation, ERAS offers a comprehensive framework that transcends surgical specialties. However, successful implementation necessitates multidisciplinary collaboration, adaptability to individual patient needs, and ongoing research to refine protocols. As medical knowledge evolves, ERAS continues to shape the landscape of modern surgery with its patient-centric, evidence-driven ethos.
